# Disruption-Induced Mucus Secretion: Repair and Protection

**DOI:** 10.1371/journal.pbio.0040276

**Published:** 2006-08-22

**Authors:** K Miyake, T Tanaka, P. L McNeil

**Affiliations:** 1 Institute of Molecular Medicine and Genetics, Medical College of Georgia, Augusta, Georgia, United States of America; 2 Faculty of Pharmaceutical Sciences, Josai University, Sakado, Saitama, Japan; 3 Department of Cellular Biology and Anatomy, Medical College of Georgia, Augusta, Georgia, United States of America; National Institutes of Health, United States of America

## Abstract

When a cell suffers a plasma membrane disruption, extracellular Ca^2+^ rapidly diffuses into its cytosol, triggering there local homotypic and exocytotic membrane fusion events. One role of this emergency exocytotic response is to promote cell survival: the internal membrane thus added to the plasma membrane acts as a reparative “patch.” Another, unexplored consequence of disruption-induced exocytosis is secretion. Many of the cells lining the gastrointestinal tract secrete mucus via a compound exocytotic mechanism, and these and other epithelial cell types lining the digestive tract are normally subject to plasma membrane disruption injury in vivo. Here we show that plasma membrane disruption triggers a potent mucus secretory response from stomach mucous cells wounded in vitro by shear stress or by laser irradiation. This disruption-induced secretory response is Ca^2+^ dependent, and coupled to cell resealing: disruption in the absence of Ca^2+^ does not trigger mucus release, but results instead in cell death due to failure to reseal. Ca^2+^-dependent, disruption-induced mucus secretion and resealing were also demonstrable in segments of intact rat large intestine. We propose that, in addition to promoting cell survival of membrane disruptions, disruption-induced exocytosis serves also the important protective function of liberating lubricating mucus at sites of mechanical wear and tear. This mode of mechanotransduction can, we propose, explain how lubrication in the gastrointestinal tract is rapidly and precisely adjusted to widely fluctuating, diet-dependent levels of mechanical stress.

## Abstract 2

Mucus secretion in response to mechanical disruption of stomach cells could explain how lubrication in the gastrointestinal tract is rapidly and precisely adjusted to widely fluctuating, diet-dependent levels of mechanical stress.

## Introduction

Plasma membrane disruption is a common form of cell injury observable under physiological conditions in a wide variety of mechanically active mammalian tissues [1]. Its occurrence was first detected in the gastrointestinal (GI) tract [2]. In mice fed membrane-impermeant microscopic markers of cell wounding, epithelial cells became labeled cytosolically in the stomach and small and large intestine. One possible cause of this “cell wounding” is gut motility, which subjects GI tract epithelia to shear and compression stresses.

The GI tract protects itself from damaging mechanical and other stresses by producing and secreting onto epithelial surfaces a layer of lubricating mucus [3]. For example, mechanical stimulation, such as distention of the stomach wall, potently stimulates mucus secretion [4]. Mucus production is, moreover, stimulated by high-fiber diets, confirming that, under physiological conditions in vivo, a mechanism is in place for sensing and responding to mechanical stress with an increase in lubrication [5,6]. However, the mechanism of this important mechanotransduction event, responsible for triggering exocytotic membrane fusion events, and consequent mucus secretion, remains unknown. We here test the novel possibility that plasma membrane disruptions, induced in mucous cells by mechanical stress, trigger protective mucus secretory events in the GI tract.

## Results

**Figure 1:**
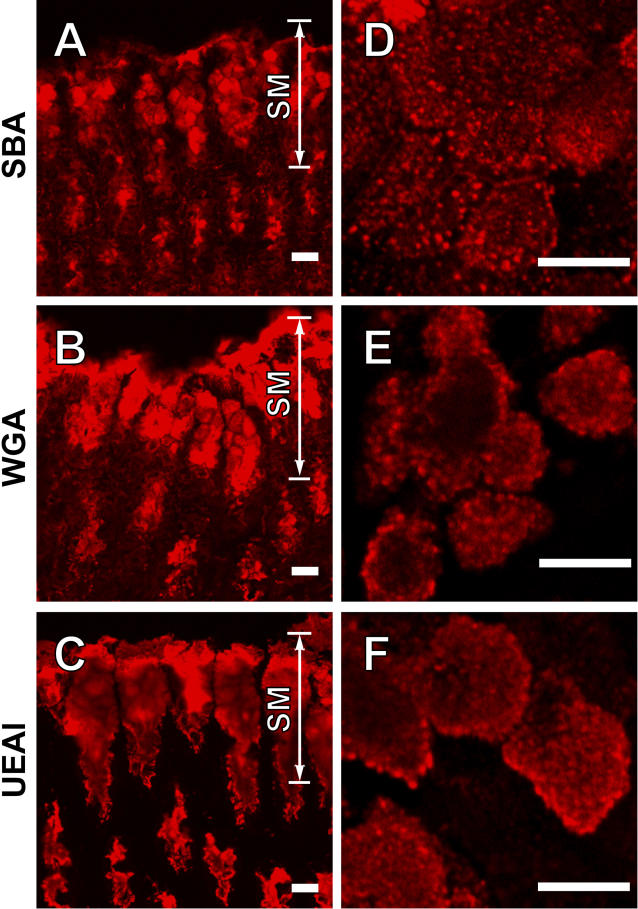
Three Lectins Strongly Stain the Intracellular Content of Gastric Surface Mucous Cells Frozen sections of the fundic portion of the mouse stomach were stained with rhodamine-labeled SBA (A), WGA (B), or UEA1 (C). Note staining of the surface mucous (SM) cells, which are the mucus-producing population of the gastric epithelium. Primary cultures of gastric surface mucous cells (asterisks) were also stained with SBA (D), WGA (E), or UEA1 (F). Confocal imaging reveals labeling of a spherical, large (~ 1-μm diameter), abundant organelle, the mucus granule. Bars represent 10 μm.

**Figure 2:**
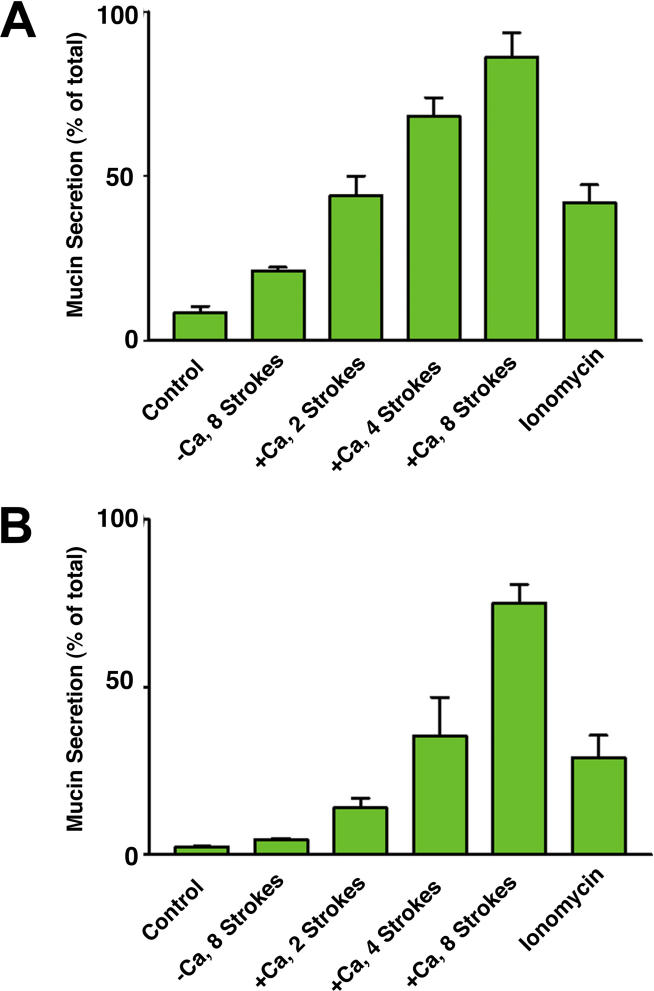
Shear Stress Induces Ca^2+^-Dependent Secretion of Mucus by Cultured Gastric Mucous Cells Cells were taken up into and ejected from (one “stroke”) a syringe operating under constant pressure in the presence of 1.5 mM Ca^2+^ (+Ca), or were not so treated (Control). One group of cells was wounded by syringing in the absence of extracellular Ca^2+^ (−Ca), and another was incubated with a Ca^2+^ ionophore (Ionomycin, 1 μM, 10 min). The medium conditioned by these cells was then harvested and the content of mucus present quantitated in an ELLA assay employing either WGA (A) or UEA (B) as the mucus ligand. All treatments, except wounding in the absence of Ca^2+^ (−Ca, eight strokes), gave mean (± standard error of the mean) values that differed significantly from the control, *p* ≤ 0.001 (A), *p* ≤ 0.01 (B), Kruskal-Wallis test.

Three different fluorescently tagged lectins, soybean agglutinin (SBA), Ulex europaeus agglutinin 1 (UEA1), and wheat germ agglutinin (WGA) all strongly stained the mucus granules of gastric surface mucous cells, both in vivo and in vitro (Figure 1), and also other mucus-producing cells (goblet cells) of the GI tract (unpublished data). These lectins were therefore employed as fluorescence markers in evaluating mucus secretion microscopically and in a quantitative enzyme-linked lectin assay (ELLA) [7] of mucus secretion. To quantitatively evaluate whether and with what level of efficacy plasma membrane disruption elicits mucus secretion, primary cultures of gastric mucous cells were wounded by syringing. Drawing suspended cells into and forcing them out of a syringe needle creates sub-lethal, reparable plasma membrane disruptions [8,9]. The medium conditioned by this cell-wounding event was evaluated for mucus content by ELLA. Increasing the number of “strokes,” or cell intake and expulsion events, resulted in a nearly linear increase in the amount of mucus present in medium, exceeding that obtainable with a Ca^2+^ ionophore (Figure 2). This assay revealed, moreover, that release failed to occur when Ca^2+^ was omitted from the medium present during syringing (Figure 2). Therefore, release of mucus was potently induced by levels of shear stress known to produce plasma membrane disruptions and was Ca^2+^ dependent.

**Figure 3:**
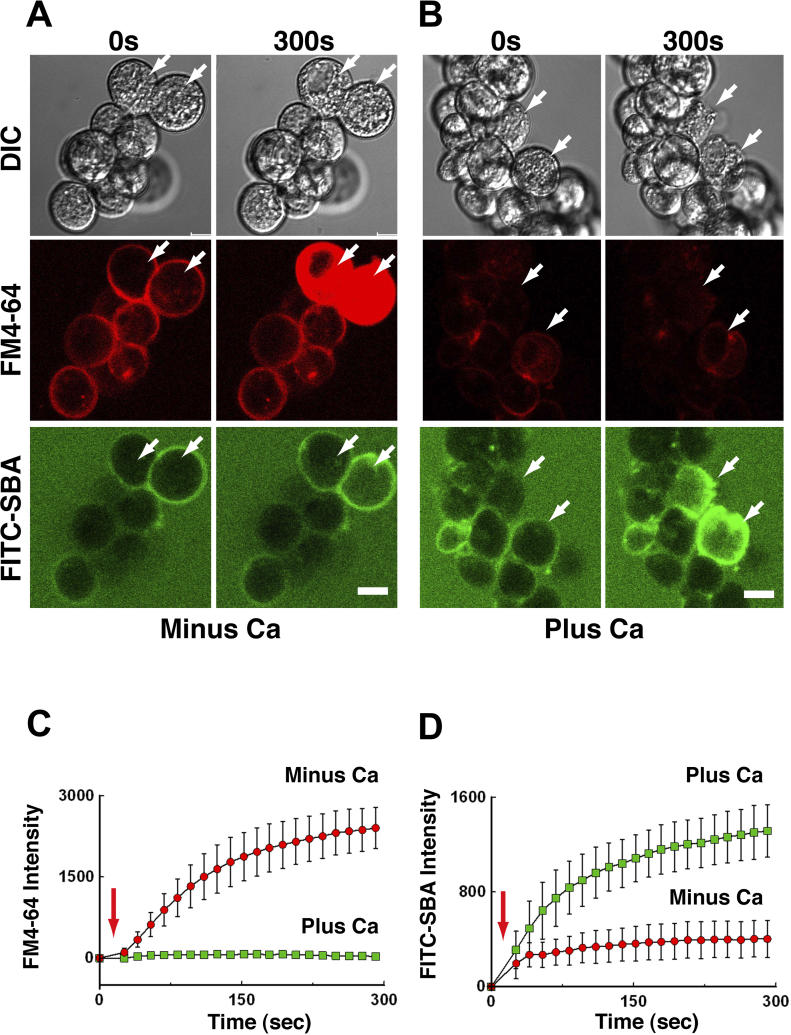
Cell Resealing and Mucus Secretion Are Ca^2+^-Dependent, Concurrent Events in Surface Mucous Cells (A) A cluster of gastric mucous cells was imaged by confocally before (0 s) and after (300 s) wounding of two cells (arrows) with a laser in the absence of Ca^2+^ (Minus Ca). Shown are differential interference contrast (DIC), and fluorescence images of FM4–64 dye (red channel), which stains the cytoplasmic membranes of cells that fail to reseal, and of FITC-SBA (green channel), which stains extracellular mucus. Note that strong cytoplasmic staining with FM4–64 is seen 300 s after wounding, whereas no detectable increase in surface staining with FITC-SBA is observed at this time point. (B) A cluster of gastric mucous cells wounded and imaged as in (A) but in the presence of 1.5 mM Ca^2+^ (Plus Ca). Note that very little cytoplasmic staining with FM4–64 is seen 300 s after wounding, whereas strong surface staining with FITC-SBA is observed at this time point. (C) Entry of FM 4–64 dye into cell cytoplasm was monitored over time after laser wounding (arrow). In the presence of extracellular Ca^2+^ (Plus Ca), a small amount of dye entry is detectable only during the first 20–30 s post-wounding, indicating that resealing was completed within this time frame. In the absence of extracellular Ca^2+^ (Minus Ca), entry of dye continues until, at approximately 100 s, internal membranes reach a saturation point. Resealing failed in these cells (*n* = 3). Bars indicate the standard error of the mean. (D) Surface staining intensity of the cells with FITC-SBA monitored over time after laser wounding (red arrow). In the presence of extracellular Ca^2+^ (Plus Ca^2+^), an increase in surface staining is detectable at 50 s post-wounding and continues throughout the time course of the experiment, indicating that, concurrently with resealing, mucus was being exocytosed. In the absence of Ca^2+^ (Minus Ca^2+^), by contrast, only a slight increase in staining was observed (*n* = 3). Bars indicate the standard error of the mean.

To test if these two events, mucus secretion and resealing, were temporally and spatially coupled responses, we imaged both simultaneously in living individual cells responding to a laser-induced plasma membrane disruption. Gastric surface mucous cells were bathed in a membrane-impermeant dye, FM 4–64, which is widely used to monitor endocytotic traffic [10]. In the short-term incubations, it strongly stains the plasma membrane only of an intact cell, but enters rapidly through an open plasma membrane disruption, where it stains the internal membrane. In the absence of Ca^2+^ (Figure 3A; Video S1), laser-wounded cells (arrows) could not restrict entry of FM4–64 dye (red channel), and consequently developed strong cytoplasmic staining with this dye over the 5-min observation period. By contrast, in the presence of Ca^2+^ (Figure 3B; Video S2), laser-wounded cells (arrows) restricted FM4–64 entry over the 5-min interval, and consequently did not develop strong cytoplasmic membrane staining. Thus, as in numerous other cell types, resealing is a Ca^2+^-dependent event in gastric mucous cells. Cells that succeeded in resealing (Figure 3B, arrows) in the presence of Ca^2+^ became, simultaneously, heavily stained with a fluorescent lectin, fluorescein isothiocyanate (FITC)-SBA (green channel), that stains extracellular mucus. Those cells (Figure 3A, arrows), by contrast, that failed to reseal due to the absence of Ca^2+^ did not develop increased surface staining with the FITC-SBA. Thus, disruption-induced mucus secretion also is Ca^2+^ dependent, and secretion occurs only in those cells that mount a resealing response. These, and additional images, were also analyzed quantitatively using image analysis (Figure 3C and 3D). Entry of FM4–64 dye was rapidly blocked in the presence of Ca^2+^ (Figure 3C), indicating that a successful resealing response had been mounted, restoring membrane integrity. This restoration of boundary formation clearly fails in the absence of Ca^2+^ (Figure 3C). During this same interval, FITC-SBA surface staining intensity rose dramatically immediately after laser wounding in the presence of extracellular Ca^2+^, but not in its absence (Figure 3D). These data directly demonstrate that Ca^2+^-triggered resealing and mucus secretion are temporally and spatially coupled in cultured gastric surface mucous cells.

**Figure 4:**
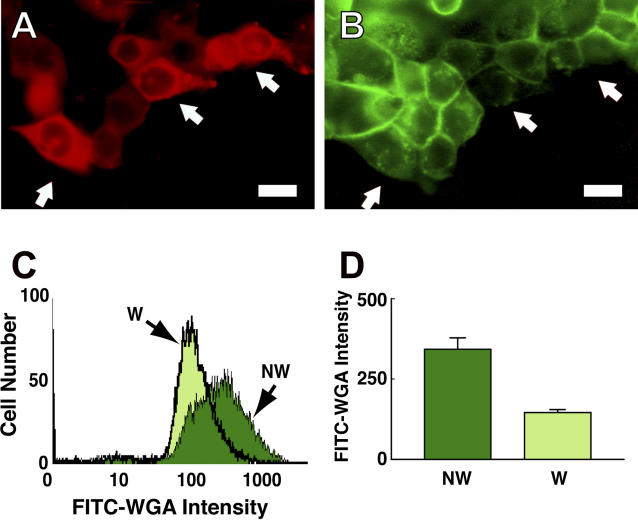
Cells That Survive a Plasma Membrane Disruption Are Depleted of Intracellular Mucus (A) MKN28 cell monolayer substrata were scratched with the tip of a needle, denuding cells and wounding many of those bordering on the denudation site. Those cells that resealed the plasma membrane disruptions that were thus created trap in their cytoplasm the Texas red-labeled (TRDx) dextran present during the scratch injury, and their cytosol is consequently fluorescent (arrows). (B) Images of the same cells after staining of intracellular mucus with FITC-WGA. Note that those cells heavily labeled with the TRDx-dextran, which suffered and repaired large plasma membrane disruptions, apparently stain more lightly with the FITC-WGA. (C) Flow cytofluorometic analysis of FITC-WGA staining in MKN28 populations that were wounded by syringing (W) or were undisturbed (NW) prior to intracellular staining of mucus with FITC-WGA. Note the downward shift in population fluorescence of the wounded relative to the undisturbed population. (D) The mean value of wounded (W) and undisturbed (NW) population fluorescence as measured by flow cytofluorometry (*n* = 3; *p* < 0.05). Bars represent 20 μm.

We next asked if loss of intracellular mucus accompanies a successful resealing response, another predicted outcome if mucus secretion is coupled to resealing. Monolayers of MKN28 cells were scratched with a sharp implement in the presence of fluorescent dextran so as to mechanically induce plasma membrane disruptions in the cells lining the scratch site [11]. Cells that survive a disruption trap the fluorescent dextran marker in their cytosol and thus can subsequently be identified [11]. The monolayers were washed thoroughly after wounding and stained after detergent permeablization (to provide access to cytoplasmic mucin) with fluorescent WGA. There was a striking negative correlation between fluorescent dextran (Figure 4A) and WGA labeling (Figure 4B), indicating that cells surviving a plasma membrane disruption had exocytosed most if not all of their mucus granules. This loss of intracellular mucus could also be demonstrated quantitatively (Figure 4C and 4D). Flow cytofluorometry was used to evaluate the intracellular staining with FITC-WGA (Figure 4C) of undisturbed MKN28 cells (dark green population) and wounded MKN28 (light green population). A downward shift (Figure 4C) in the fluorescence of wounded population (light green) relative to the undisturbed population (dark green) was reproducibly observed (Figure 4D).

**Figure 5:**
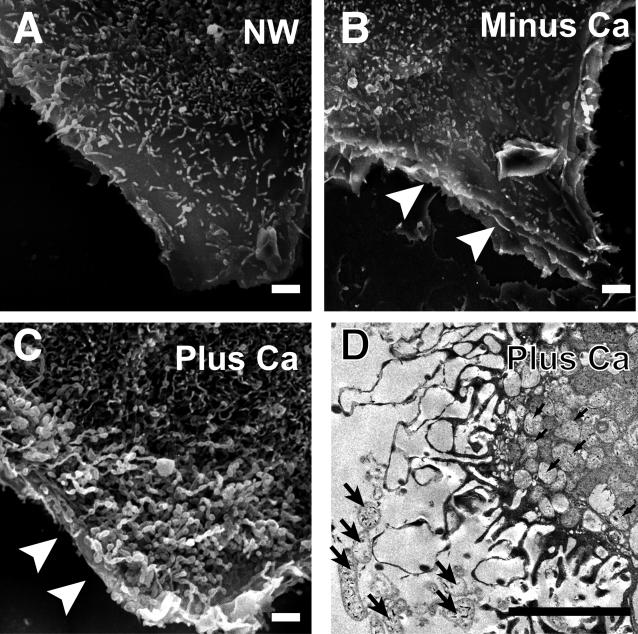
Mucus-Producing Cells Exhibit a Ca^2+^-Dependent Change in Surface Architecture following Wounding Subconfluent monolayers of cultured MKN28 cells were wounded by scratching the glass substratum with a needle tip, and then prepared for scanning electron microscopy. (A) Scanning electron micrograph of an undisturbed cell (NW) not bordering on a scratch site. (B) Scanning electron micrograph of a cell bordering on a scratch site made in the absence of extracellular Ca^2+^ (Minus Ca). The arrowheads mark a site of plasma membrane discontinuity, presumably a disruption that failed to reseal. (C) Scanning electron micrograph of a cell bordering a wound site made in physiological Ca^2+^ (Plus Ca). Arrowheads mark the presumptive plasma membrane disruption site. Note the dramatic difference (from cells in [A] and [C]) in surface architecture adjacent to this presumptive membrane disruption site, which includes numerous villus-like projections of membrane, and spherical profiles, presumably the exocytosed content of mucus granules. (D) Transmission electron micrograph of a cell bordering on a wound site made in physiological Ca^2+^. Entry of horseradish peroxidase, present extracellularly while making the scratch, results locally in the prominent dark labeling of villi and other sub-plasma membrane spaces, and marks this location as one at or nearby the disruption site. Note the numerous, microvillar extensions and the presence of mucus granules both within the cell (arrows) and decorating its surface. Bars represent 2 μm.

The massive deposition of cytoplasmic membrane onto surface plasma membrane that accompanies mucus secretion [12] would be expected to dramatically alter surface architecture. To test this prediction, we used scanning electron microscopy (SEM) to compare the surface morphology of undisturbed MKN28 cells (Figure 5A) with that of cells wounded in the absence of Ca^2+^ (Figure 5B) or in the presence of resealing-permissive physiological Ca^2+^ (Figure 5C). Wounding in the presence of Ca^2+^ (Figure 5C) induced a dramatic alteration in surface architecture: the cell becomes richly decorated with numerous membrane protrusions, as expected if disruption-induced exocytosis had added additional membrane to the surface. Additionally, spherical structures, possibly representing exocytosed mucus granules, were observed on the surfaces of wounded cells. Transmission electron micrographs (Figure 5D), through disruption sites of cells wounded in the presence of Ca^2+^, confirmed that a dramatic alteration in surface architecture accompanies resealing, and that this alteration involved the formation of microvilli and liberation of mucus granules (intracellular granules are marked by arrows) onto the cell surface.

**Figure 6:**
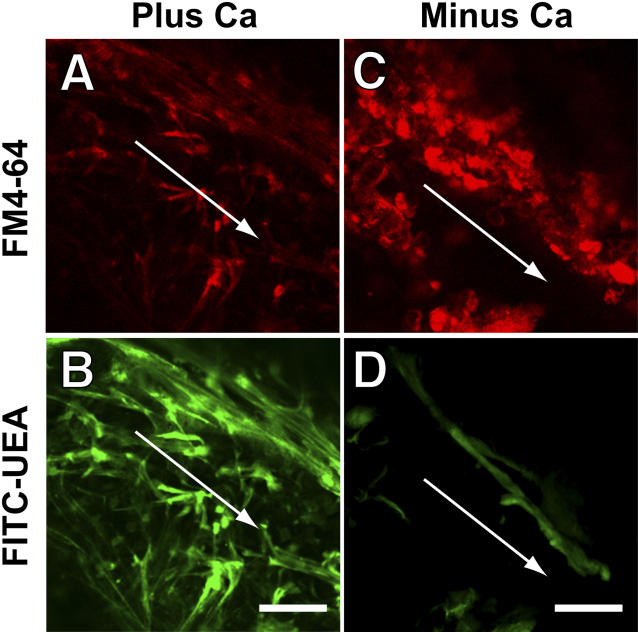
Ca^2+^-Dependent Secretion of Mucus, and Resealing, Can Be Observed in an Excised Segment of Colon A segment of excised colon was scratched (the arrows mark the approximate sites) in the presence (Plus Ca) and absence (Minus Ca) of physiological Ca^2+^. FM4–46 dye, a marker for cells that failed to reseal, and FITC-UEA, a marker of surface mucus, were added 3 min later. Confocal images of the segment were then immediately acquired of the FM4–64 (A) and (C) and FITC-UEA (B) and (D) staining. As indicated by relative FM4–64 staining intensities, an increased incidence of resealing failure occurred in the absence of Ca^2+^. As indicated by the relative FITC-UEA staining intensities, on the other hand, a dramatically decreased incidence of mucus secretory events occurred in the absence of Ca^2+^. Bars represent 50 μm.

Finally, we asked if disruption-induced mucus secretion could occur in an excised segment of the GI tract. A segment of rat colon was immersed in saline with or without added Ca^2+^, scratched with a sharp implement, and then exposed to saline containing FM 4–64 and FITC-UEA. When the scratch was carried out in the presence of Ca^2+^, staining with FM 4–64, which strongly stains cells that fail to reseal (see above), was minimal (Figure 6A), whereas strong staining with the mucus stain, FITC-UEA, was observed (Figure 6B). The converse staining pattern was observed in the absence of Ca^2+^ (Figure 6C and 6D). Thus, membrane disruptions made in intact segments of gut under resealing-permissive conditions, as in the in vitro models systems described above, also result in a strong, local mucus secretory response, one that is coupled to a resealing response. Resealing failure, on the other hand, is associated with minimal mucus release.

## Discussion

Mucin, in vivo, is stored in a dense cluster of apical granules, both in the surface mucous cell which continuously lines the entire luminal surface of the stomach, and in the specialized goblet cells, which are intermittently present throughout small and large intestine epithelia. Secretion apparently can occur constitutively, but it also clearly can be regulated [13]. Given the protective role of mucus, it is not surprising that numerous injurious chemical agents stimulate mucus secretion, as do mechanical stimuli.

Several neurohormonal agonists capable of triggering mucus secretion have been identified. During secretagogue-induced mucin secretion, both homotypic (between mucin granule membranes) and heterotypic/exocytotic (between granule and plasma membranes) fusion events occur, a process referred to as *compound exocytosis* [12]. The result is that a single, large bolus of mucus, the contents of many fused mucus granules, is secreted. Candidate secretagogues include cholinergic agents, which can activate cells of salivary glands, and the cultured mucous cells of the stomach and small intestine goblet cells, but only those present in the crypts. Interestingly, given the potential importance of direct mechanical regulation for those cells directly exposed to luminal stresses, surface- and villus-resident mucous cells become refractory to neurohormonal stimuli [14]. Neuropeptides also will induce mucus secretion from certain mucous cell lines in vitro, and prostaglandins induce mucus secretion from cultured cells, cell lines, and gastric surface mucous cells [3,7,15]. However, the physiological significance of these and other described chemical agonists remains unclear.

Regulated secretion of mucus strikingly resembles membrane fusion-based resealing. In both, a rise in cytosolic Ca^2+^ [16–19] can trigger homotypic and heterotypic membrane fusion events leading to the massive surface deposition of internal membrane, a process referred to as compound exocytosis in relation to mucus secretion [12] and patching in relation to resealing [20]. Strikingly, secretagogue-stimulated mucus secretion in vivo is accompanied by the sloughing of considerable cytoplasmic constituents, and also of membrane, into the extracellular milieu [12]. This is true also for resealing-based exocytotic fusion [20]. The loss of cytoplasm just mentioned strongly implies that, in fact, during secretagogue-stimulated secretion, a disruption in plasma membrane continuity occurs, and that, therefore, all strong mucus secretory events are, in part, a resealing response. Such sloughing of unused membrane may be the result of a “vertex” fusion mechanism [21]. In this mode, rather than a single fusion pore, membranes are envisioned to interact extensively along vertices [22]. The predicted result, when this model is applied to a resealing or compound exocytotic event, is the excision, and consequent sloughing, of a large segment of the fusing membrane. Vertex fusion may, therefore, be another shared characteristic.

**Figure 7:**
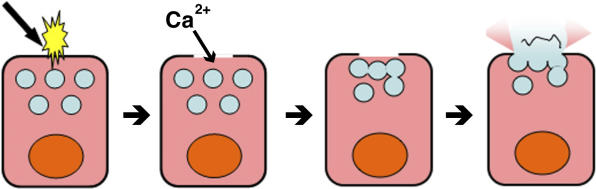
Coupling of Repair and Protective Mucus Secretion Mechanical or other stressors produce a plasma membrane disruption in a mucous cell lining the GI tract. Ca^2+^ enters through the disruption. Homotypic fusion of granules with one another is triggered by this signal. Exocytotic fusion of the homotypic fusion product with the plasma membrane surrounding the defect site completes repair and liberates mucus into the extracellular environment. Some cytoplasmic components and membrane are also liberated (see text for explanation).

We here propose that, in mucus-producing cells of the GI tract, plasma membrane disruption events, which occur physiologically in this mechanically active environment, potently induce mucus release. This adaptation to a stressful environment would confer two benefits. First, the wounded cell survives the disruption. Our results suggest that the cytoplasmic membrane compartment utilized for repair is not, as has been previously proposed for fibroblasts and other cultured cells, the lysosomal [23] or endocytotic [24,25] compartments but instead, a classical secretory (mucus granule) pathway compartment. The positioning of this compartment, at cellular apices exposed to injurious events, may indeed represent an adaptation that facilitates rapid repair in a particularly hostile environment. Second, and perhaps more important, a deposit of lubricating mucus is made at sites of mechanical wear and tear, which could then serve to protect other cells from injury. Strongly supporting this hypothesis is our finding in three different mucus-producing cell types––primary cultures of surface gastric mucous cells, a cultured cell line, and intestinal goblet cells––that a plasma membrane disruption can potently induce mucus secretion. Release of mucus is, in each case, not simply a matter of cell lysis, which is far more effectively achieved when Ca^2+^ is absent and resealing is therefore blocked. Rather, as we show here by monitoring these events simultaneously in living, single cells, mucus secretion and successful resealing occur simultaneously, and both require that external Ca^2+^ be present during a plasma membrane disruption. Ca^2+^ entry through a plasma membrane disruption, we propose, involves mucus granules in a homotypic fusion response leading to the creation of a patch vesicle, or compound exocytotic granule, from individual granule membranes (summarized schematically in Figure 7). Its exocytotic annealing to surrounding plasma membrane then reseals the disruption, and, most important, deposits a bolus of mucus on the cell surface.

Mucus secretion by this mechanism might protect against forms of injury other than mechanical. For example, when the rodent stomach is insulted with injurious levels of ethanol, a protective layer of mucus is rapidly laid down, under which locomotion by cells protected in glandular epithelial invaginations (gastric pits) rapidly promotes restitution of epithelial integrity [26]. Our hypothesis suggests that protection does not require that the secreting mucus cells necessarily survive an insult, but rather that release of mucus occur, e.g., that it be liberated from its cytoplasmic, membrane-bound state. For this to transpire, all that would be required is extracellular Ca^2+^. Two sources are available in the stomach. One is saliva, which is notably rich in Ca^2+^ [27] and, hence, represents a constitutive source of extracellular Ca^2+^ for surface epithelial cells. A second source, available apically as a signal for mucus-producing cells, but only upon injury, is the Ca^2+^ present in the abluminal, extracellular environment. Abluminal fluids rapidly permeate any paracellular route opened by an injury to the epithelium, such as an ethanol insult [26]. We propose that wounds to mucus-producing cells, occurring under physiological or pathological conditions, and caused by either chemical or physical agents, result in an important adaptive response in the GI tract, the elaboration of a protective layer of mucus. Onsite mucus lubrication is thus rapidly and directly tuned to the widely fluctuating stress levels of the GI environment.

## Materials and Methods

### Cells and tissue.

Surface gastric mucous cells were isolated as previously described [7]. Briefly, the stomach was removed from an anesthetized rat, everted, inflated with Hank's buffer, and then immersed in dispase (50 U/ml; BD Biosciences, San Jose, California, United States) for 1 h at 37 °C. Surface mucous cells were dislodged by gentle tituration of the everted surface in Hank's containing 0.1% BSA and 0.5 mM EGTA, and were filtered through nylon mesh (150 gauge) prior to culture in 35-mm dishes on a collagen gel (Type 1, rat tail; BD Biosciences). Cells were harvested for experimentation 2–3 d later by trypsinization. MKN28 cells (the gift of H. Hojo, Fukushima Medical University) were cultured to confluence in RPMI 1640 medium containing 10% calf serum as originally described [28]. In some cases, a segment of the large intestine was removed, split longitudinally, and then washed thoroughly in PBS. It was then scratched with a 22-gauge syringe needle in PBS containing 1.5 mM Ca^2+^ or no added Ca^2+^.

### Electron microscopy.

Scanning electron microscopy [29] and transmission electron microscopy [30] were performed as previously described.

### Flow cytometry.

Samples of 10,000 cells were analyzed by flow cytometry (Becton Dickinson FACscan system, BD Biosciences) at an excitation wavelength of 488 nm and an emission bandpass of 530 nm, as previously described [30].

### Syringe wounding.

Syringing was carried out as previously described [9] on a semi-automated device, at a constant setting of 40 PSI, capable of reproducibly exposing cells to shear stress by drawing them up into and expelling them from a syringe needle (30 gauge). One “stroke” is defined as one uptake and expulsion event applied to the cell population.

### Laser wounding and image analysis.

Trypsinized surface mucous cells seeded into 35-mm dishes were subject to laser wounding at 37 °C in the presence of FM4–64 dye (2.5 μg/ml; Molecular Probes, Eugene, Oregon, United States) and FITC-SBA (10 μg/ml; Vector Laboratories, Burlingame, California, United States), and dye uptake and lectin fluorescence monitored by time-lapse image acquisition and image analysis as previously described [31] on a multi-photon microscope (Zeiss LSM510 Meta, Zeiss, Oberkochen, Germany).

### Plate reader assay of mucus secretion.

Mucus release was quantified as previously described [7] using ELLA. Briefly, following syringe wounding, cells and debris were separated from the conditioned medium by centrifugation (800 rpm for 3 min). Soybean agglutinin (25 μg/ml, 3–5 h, 21°C) was used to coat the bottoms of 96-well plates, which were then washed with buffer consisting of 0.1% BSA and 0.05% Tween20 in PBS. Following an overnight incubation with samples or standards (purified rat gastric mucin, 15–1,000 ng/well), the samples were rinsed with wash buffer, and biotinylated UEA (1 μg/ml, 2 h, 21 °C) was added. Finally, biotin levels were detected, following further washing, using a standard kit (Vectastain ABC, Vector Laboratories) and a plate reader (Spectra Max 250, Molecular Devices, Menlo Park, California, United States).

## Supporting Information

Video S1 Movie (QuickTime) Illustrating the Typical Resealing and Mucus Secretory Responses of Gastric Mucous Cells Wounded by Laser Irradiation in the Absence of Extracellular Ca^2+^
Plasma membrane disruptions were created with a laser in two gastric mucous cells (arrows mark the site) in the second frame of this time-lapse series (frame interval = 15 sec, 5 min total) of confocal images. FM4–34 dye (red stain) begins immediately to enter through these disruptions, and this entry occurs throughout the time course of this movie. Resealing by these two cells fails. FITC-SBA present in the medium (green stain) initially stains the surface of the wounded cells lightly, but this staining does not increase after wounding. These wounded cells also fail to secrete mucus.
(1.4 MB MPG)

Video S2 Movie (QuickTime) Illustrating the Typical Resealing and Mucus Secretory Responses of Gastric Mucous Cells Wounded by Laser Irradiation in the Presence of Extracellular Ca^2+^
Plasma membrane disruptions were created with a laser in two gastric mucous cells (arrows mark the site) in the second frame of this time-lapse series (frame interval = 15 sec, 5 min total) of confocal images. FM4–34 dye (red stain) begins immediately to enter through these disruptions, but in this case, entry rapidly ceases, as evidenced by staining with this dye. Resealing occurred in these two cells. FITC-SBA present in the medium (green stain) initially stains the surface of the wounded cells, and this staining increases dramatically following wounding. These wounded cells also secreted mucus.
(1.3 MB MOV)

## Abbreviations

ELLA: enzyme-linked lectin assay
FITC: fluorescein isothiocyanate
GI: gastrointestinal
SBA: soybean agglutinin
WGA: wheat germ agglutinin
UEA: Ulex europaeus agglutinin
